# 

**Published:** 1885-03

**Authors:** 


### CONTENTS:

**Art. I—Proceedings of Chicago Dental Society..........481**
**Art. II—Symptomatic Pathology of the Mouth**
**By Elton R. Smilie, M. D..........494**
**Art. III—A History of the work "Principles and Practice of Dentistry"..........505**
**Art. IV—At What Age Teeth Should Have the Most Attention.**
**By Geo. A. Mills, D. D. S..........512**
**Art. V—Kansas State Dental Laws..........515**
**Art. VI—National Association of Dental Examiners..........518**

### EDITORIAL, ETC.

**The International Medical Congress..........519**
**General Grant's Disease..........520**

### MONTHLY SUMMARY.

**Removal of Stains from Teeth..........521**

**Choice of Teeth for Extraction for the purpose of Uniformity..........524**

**Japanese Teeth..........524**

**The Hypodermic Syringe, Manner of Using..........525**

